# Proposing magnetoimpedance effect for neuromorphic computing

**DOI:** 10.1038/s41598-023-35876-0

**Published:** 2023-05-27

**Authors:** Loghman Jamilpanah, Alessandro Chiolerio, Marco Crepaldi, Andrew Adamatzky, Majid Mohseni

**Affiliations:** 1 Department of Physics, Shahid Beheshti University, Evin, Tehran, 19839 Iran; 2 Laboratory for High Performance Ceramics, Empa - Swiss Federal Laboratories for Materials Science and Technology, 8600 Dübendorf, Switzerland; 3 Bioinspired Soft Robotics, Center for Converging Technologies, Istituto Italiano di Tecnologia, Via Morego 30, 16165 Genoa, Italy; 4 Electronic Design Laboratory, Center for Human Technologies, Istituto Italiano di Tecnologia, Via Enrico Melen 83, 16152 Genoa, Italy; 5 Unconventional Computing Laboratory, University of the West of England, Coldharbour Lane, Bristol, BS16 1QY UK

**Keywords:** Applied physics, Electronics, photonics and device physics, Materials for devices

## Abstract

Oscillation of physical parameters in materials can result in a peak signal in the frequency spectrum of the voltage measured from the materials. This spectrum and its amplitude/frequency tunability, through the application of bias voltage or current, can be used to perform neuron-like cognitive tasks. Magnetic materials, after achieving broad distribution for data storage applications in classical Von Neumann computer architectures, are under intense investigation for their neuromorphic computing capabilities. A recent successful demonstration regards magnetisation oscillation in magnetic thin films by spin transfer or spin orbit torques accompanied by magnetoresistance (MR) effect that can give a voltage peak in the frequency spectrum of voltage with bias current dependence of both peak frequency and amplitude. Here we use classical magnetoimpedance (MI) effect in a magnetic wire to produce such a peak and manipulate its frequency and amplitude by means of the bias voltage. We applied a noise signal to a magnetic wire with high magnetic permeability and owing to the frequency dependence of the magnetic permeability we got frequency dependent impedance with a peak at the maximum permeability. Frequency dependence of the MI effect results in different changes in the voltage amplitude at each frequency when a bias voltage is applied and therefore a shift in the peak position and amplitude can be obtained. The presented method and material provide optimal features in structural simplicity, low-frequency operation (tens of MHz-order) and high robustness at different environmental conditions. Our universal approach can be applied to any system with frequency dependent bias responses.

## Introduction

“Neuromorphic computing might be the answer to the hardware problem of artificial intelligence”, says a recent editorial in Nature Electronics^1^. Dynamical phenomena and oscillations synchronisation covers a prominent portion of neuromorphic computing^2–5^. Design and implementation of physical systems and materials, capable of emulating these oscillations and thus permitting their use for computing tasks, has been the subject of many researches in recent years^6^. Magnetic materials may be one of the most promising competitors for neuron-based computing due to their high stability, retention, energy efficiency and miniaturisation potential which have already resulted in their implementation in computer memories^7,8^. Nowadays, they have been used to perform neuromorphic computing tasks based on oscillation of magnetisation^9,10^. Their successful implementation has been achieved thanks to their oscillation frequency tunability obtained through an applied bias current passing through them. The magnetisation oscillation together with MR effect cause the voltage/current oscillation^9–11^. Despite such appealing features, operating thin films and achieving the conditions for a stable micromagnetic structure and controllable spin-torque effect requires the fabrication of ultra-thin structures with planar interfaces without local trapping states and impurities^12^, the achievement of high spin injection efficiency^13^, and more generally the use of dedicated equipment^14^.

**Figure 1:**
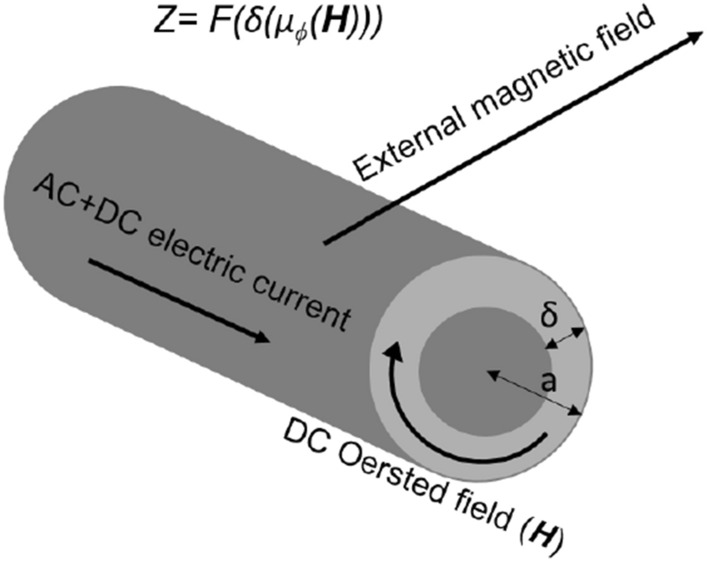
Schematic of an AC electric current passing through a magnetic wire with circular permeability $\mu _\varphi$. The impedance can be changed by external magnetic fields submitted to the system through controlling the $\mu _\varphi$ [this functionality can be written as $Z= F(\delta (\mu _\varphi (\textbf{H})))$].

Here we present magnetic permeability characteristic of magnetic wires for the exploitation of a peak in the frequency spectrum of the voltage and enable its control through a bias voltage. The physical basis is the frequency dependence of permeability which results in frequency dependence of impedance through the classical skin effect^15^. According to Maxwell theory, when a current with frequency *f* passes through a magnetic material with transverse magnetic permeability $\mu$ (perpendicular to the direction of passing current), the impedance is given by,

$$\begin{aligned} Z = \frac{R_{DC} k a J_0(ka)}{2J_1(k a)}, \end{aligned}$$

where $k= (1+i)/\delta$ and $J_0$ and $J_1$ are the Bessel functions of first type, *a* is the radius of the wire, $\delta$ is the magnetic penetration depth, given by,

$$\begin{aligned} \delta = c \,(8\pi \sigma \mu _\varphi f)^{-1/2}, \end{aligned}$$

with $\sigma$ electrical conductivity, *f* frequency of the current along the sample and $\mu _\varphi$ is the circular magnetic permeability^16,17^. Figure 1 shows a schematic of the effect where magnetic fields presented in the system, including Oersted field (**H**), can change the transverse magnetic permeability, and therefore the skin depth, and as a result the impedance can change. The effect is known as magnetoimpedance (MI) effect and has been well-known since many years^17^.

Here, for the first time, we apply an electrical noise signal to a magnetic wire and look at the voltage amplitude of the wire in the frequency spectrum. We observe a peak in the spectrum with frequency and amplitude tunability under an applied DC bias voltage thanks to the MI effect. DC bias current creates a DC tangential magnetic field, which affects the circular permeability and consequently the impedance of the wire. Significant dependence of the impedance on DC bias current has been observed already^18,19^. The observed peak is due to the maximum of the permeability at the peak position. Generally, in magnetic materials, $\mu$ increases up to a specific *f* by increasing domain wall speed and domain rotation. By further increase of *f* the $\mu$ decreases due to damping of domain wall motion by Eddy currents^17^. By applying an external bias voltage, different frequencies give different MI ratios and result in a net shift of the peak frequency and amplitude. Nonlinear evolution of the amplitude and frequency of the peak is observed by applying a DC bias voltage. The simple electrical control over the peak can be used for training a neural network composed of some magnetic wires implemented in a circuit. The lower functioning frequency and simple structure of the presented method feature some advantages in comparison to typical spin torque oscillators which operate at higher frequencies^9–11^. Also, the possibility of manipulating the impedance of these wires through spin-orbit torque (SOT), according to our previous demonstration^20^, is promising for the successful implementation of our proposed computing method for tasks with efficient energy consumption.

## Materials and methods

**Figure 2:**
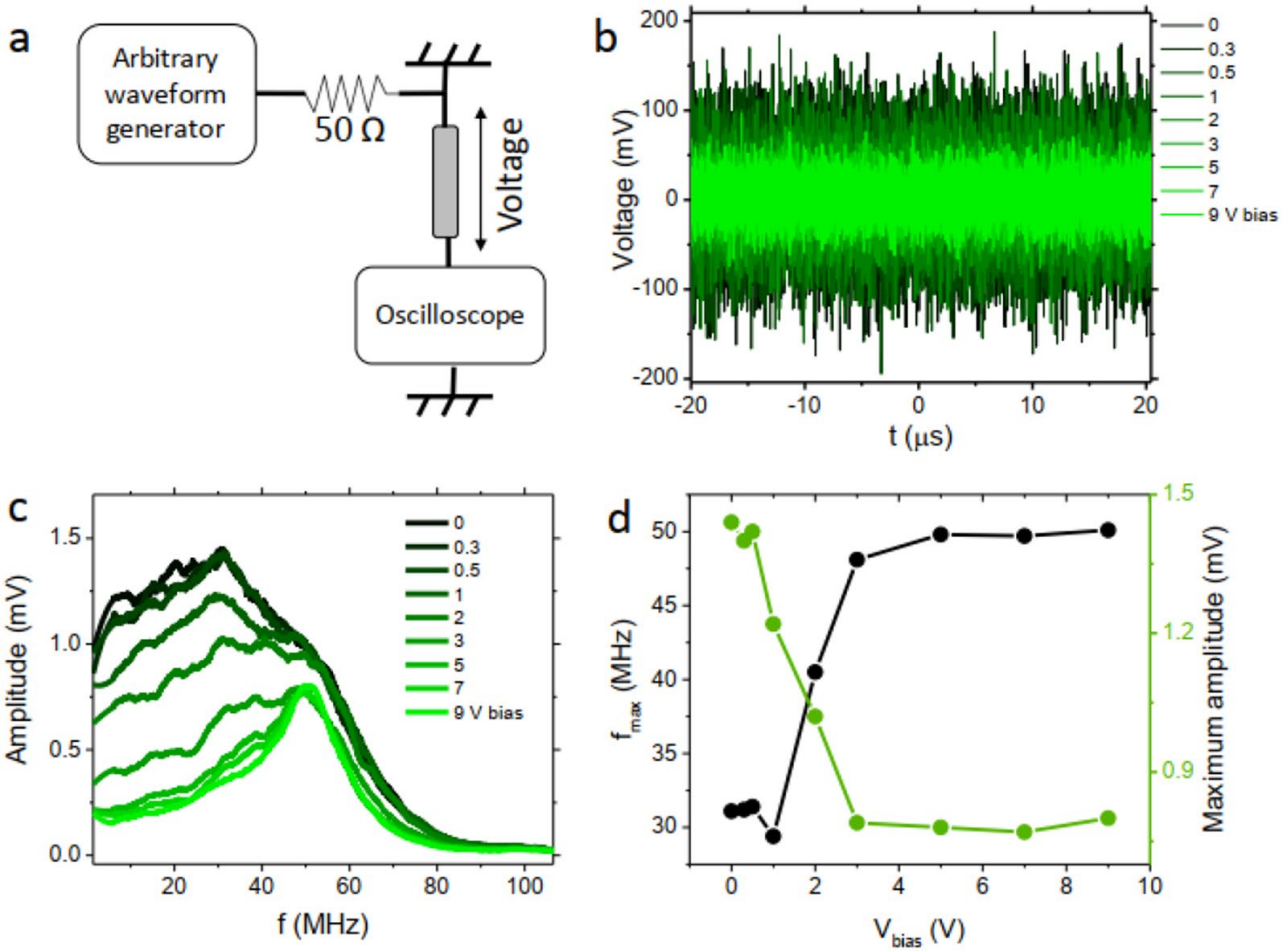
(**a**) Circuit used for the creation of the peak at the frequency spectrum of the voltage using a magnetic wire. (**b**) Measured noise signal from the oscilloscope. (**c**) The FFT of the measured noise signal under different applied voltage biases. (**d**) Frequency and amplitude of the produced peak versus applied bias voltage.

Amorphous Co-based wire with the composition of ${\textrm{Co}}_{68.15}{\textrm{Fe}}_{4.35}{\textrm{Si}}_{12.5}{\textrm{B}}_{15}$ was purchased from metallurgy laboratory of Sharif University. For the electrical measurements a straight wire with 40.0 mm length and $\approx$110 $\upmu$m diameter is used. The voltage signals are applied in the longitudinal direction of the wire using a function generator (GPS-2125), with a 50 $\Omega$ resistor series in the circuit. The voltage across the wire is measured using a digital oscilloscope (GPS-1102B). AC coupling mode of the oscilloscope is used in the measurements for convenience in the data recording. The schematic of the measurement setup can be seen in Fig. 2a.

## Results

### Dependence of the frequency and amplitude of the peak to the bias voltage

Measurements outcomes when noise and DC bias voltage are applied to the magnetic wire are presented in Fig. 2. The amplitude of the noise is chosen to have 0.1 V variance. Figure 2b shows the voltage signal saved from the oscilloscope. Clearly, the variance of the noise signal decreases by increasing the bias amplitude. For detailed analysis of the voltage amplitude at each frequency the fast Fourier transform (FFT) signal is derived and depicted in Fig. 2c. For clarity the FFT signal is smoothed by averaging 500 points of the plot. The amplitude shows a maximum at about 30 MHz with zero bias voltage and by increasing the bias voltage to 9 V the peak frequency shifts to 50 MHz. The bumpy amplitude of signal at low voltage bias can be justified by the randomness of the magnetization which has a very high permeability and easily can be affected by environmental signals^16,21^. Impedance of the wire increases by increasing the frequency up to a specific point (30 MHz here), and by further increasing frequency the domain wall movement is damped and the permeability/impedance decreases consequently. It can be seen that by applying bias voltage the amplitude decrease mainly occurs at lower frequencies which leads to the peak shift towards higher frequencies. Therefore, frequency dependency of MI effect plays an important role in the obtained result. Also, the observed shift is affected by the impedance of the used circuit elements (for a broader discussion see section comparison with nonmagnetic wires). Another notable change is the decrease of the peak amplitude by increasing the voltage bias. In Fig. 2d the evolution of both frequency and amplitude of the peak versus voltage bias is presented. As it can be observed, the peak voltage and frequency change are nonlinear and the response is similar to those measured in STOs^10^. Besides, we have previously demonstrated that the impedance can be a function of previous magnetization states when the wire is covered with cobalt^22^, acting as a memory. Upon decrease of the voltage the original FFT signal can be restored. This cycle can be repeated for many times without losing the properties of the observed signal. This stability gives a substantial advantage, compared to the current technologies, for producing ultra stable neuromorphic devices. MI effect of ${\textrm{Co}}_{68.15}{\textrm{Fe}}_{4.35}{\textrm{Si}}_{12.5}{\textrm{B}}_{15}$ amorphous wires has been investigated intensely due to their high magnetic permeability (at the order of 104 $\mu _0$^23^, $\mu _0$ is the magnetic permeability of the vacuum) which results in high MI ratios (at the order of $\approx$102%^24^). The resistivity of the used material has been reported to be 130 $\upmu \Omega \,{\textrm{cm}}$^25^. In the following, we discuss some aspects of the observed phenomena which can be considered for better understanding of the underlying mechanisms and also their further possible developments.

### Higher harmonics effects

**Figure 3:**
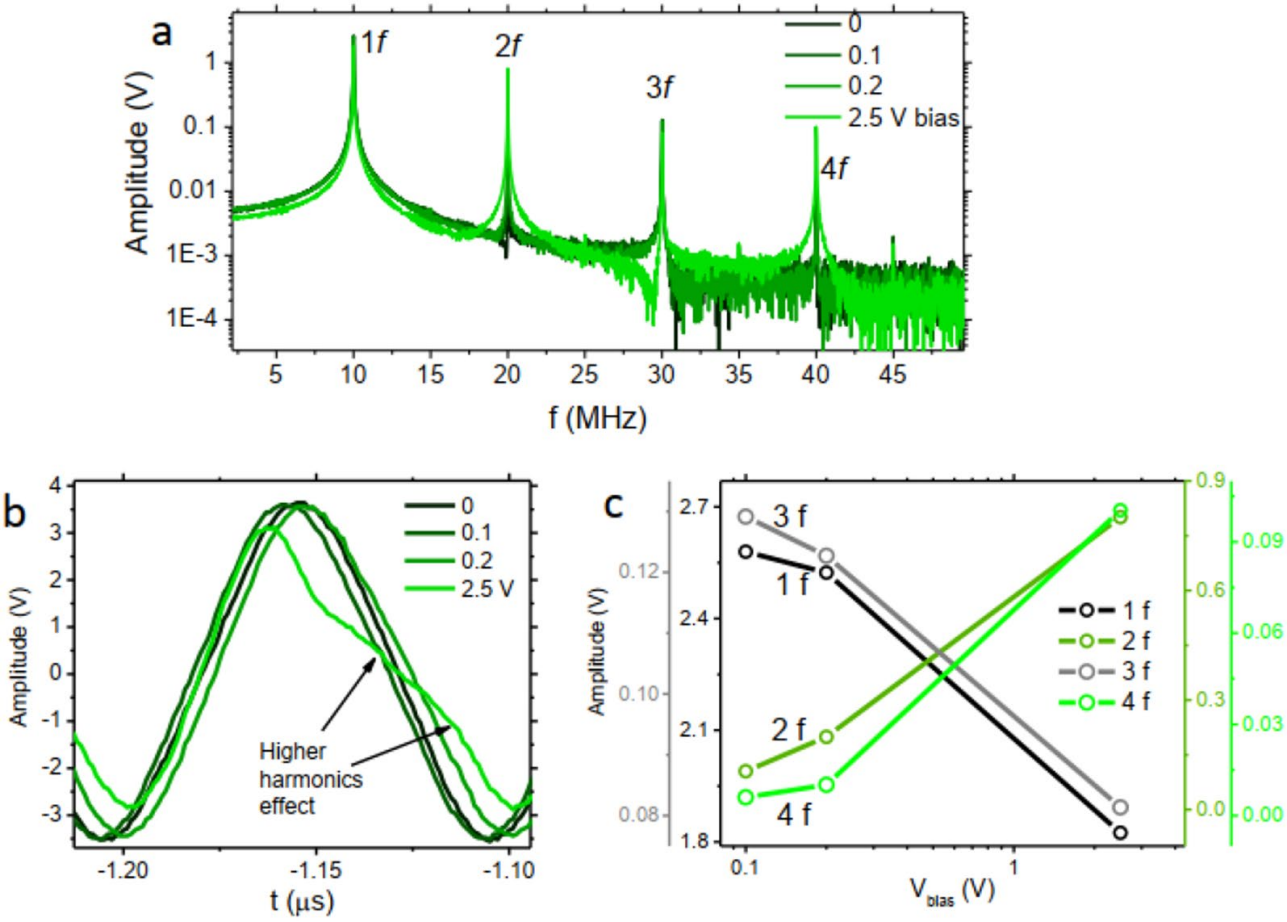
(**a**) FFT signal of the magnetic wire when 10 MHz AC voltage is applied to the wire with different voltage biases. (**b**) The measured voltage signal from the oscilloscope under different bias voltages. (**c**) The higher harmonics voltage amplitude versus applied voltage bias.

In the observed frequency spectrum of the voltage not only the first harmonic but also higher harmonics might play a role. To investigate how higher harmonics might play a role in the frequency and amplitude shift of the peak, we present measurements for a fixed frequency. When the AC magnetic field is in the order of, or larger than, the anisotropy field, nonlinear effects can be observed in the MI signal^26^. Odd harmonics are always present in nonlinear MI and even harmonics are present in wires with helical anisotropy, where asymmetry in the circumferential hysteresis loop is present^27–29^. Here we apply a 10 MHz AC signal with large amplitude (10 $V_{\textrm{pp}}$) to the circuit of Fig. 3a. Figure 3b shows the voltage signal where higher harmonic effects can be observed through deviation from sinusoidal form when bias voltage increases. Figure 3a is the FFT signal of the data showing higher harmonics peaks. By taking a closer look at the evolution of the harmonics amplitude (Fig. 3c) it can be seen that amplitude of odd/even harmonics decreases/increases by increasing bias voltage. This trend has been theoretically predicted for wires with transverse magnetic anisotropy^30^ and here we observe it experimentally, for the first time. One notable characteristic of the higher harmonics is the much higher sensitivity of the even harmonics to the bias voltage. The amplitude change of 4f harmonic in Fig. 3c corresponds to $\approx$ 1500$\%$ change in the amplitude of this harmonic. The higher sensitivity of higher harmonics has been observed by others as well^25^. This might provide an opportunity for larger range frequency and amplitude peak shifts in the frequency spectrum. The presence of higher harmonics in the system may give the possibility of featuring larger FFT peak frequencies and amplitude tunability ranges. There is room to further investigate the higher harmonics effects when the noise is applied with different applied amplitudes of the frequencies. A simulation demonstration of the harmonics can be seen in supplementary information.

### Comparison with nonmagnetic wires

**Figure 4:**
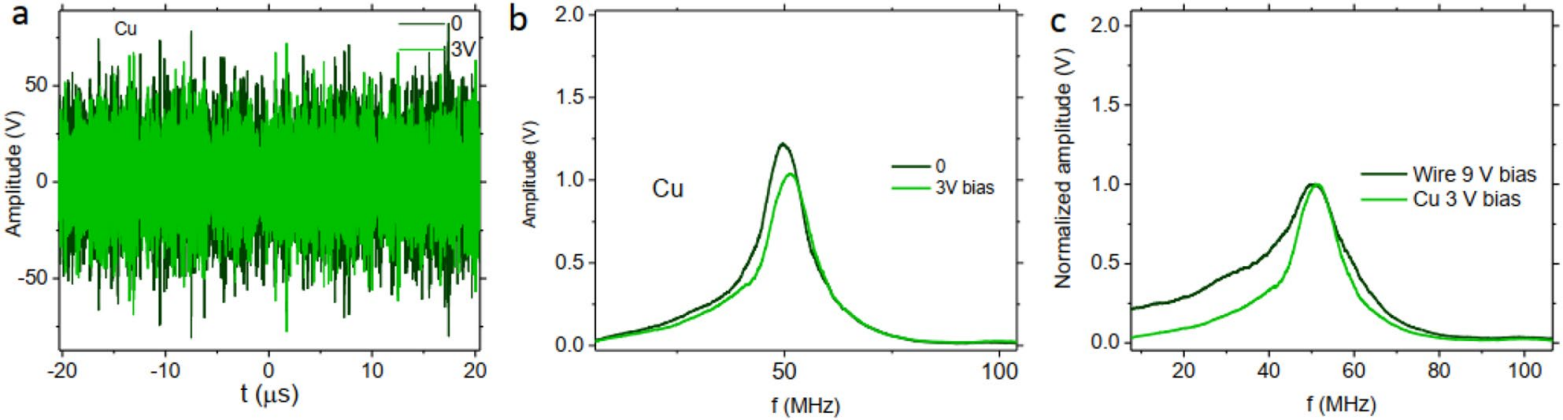
(**a**) Oscilloscopes measurement for the nonmagnetic Cu wire under applied noise and bias voltage, (**b**) FFT signal of the Figure 4a signals, (**c**) Comparison of the normalized FFT voltage signal of magnetic wire and nonmagnetic Cu wire.

To see the effect of the whole circuit on the observed signals in our experiment we repeated the measurements using a nonmagnetic Cu wire with similar geometry (115 $\upmu$m diameter and 4 cm length). In Fig. 4a we can observe that the voltage signal of the Cu wire under bias voltage shows a very small change which might be due to the change in the induction of the wire. Also a slight difference in the FFT signal can be appreciated in Fig. 4b. It can be seen that the measured FFT signal of the Cu wire is similar to the magnetic wire when the highest bias voltage is applied. Figure 4c compares the normalized FFT signal of the magnetic wire and of the Cu wire at the saturation condition (highest bias voltage). This plot clearly indicates that the saturated position is a characteristic of the circuitry used and also of the noise source. Results indicate the importance of the used circuitry elements for the final peak position and amplitude. Also results of measurement on nonmagnetic Cu wire show the importance of using a magnetic wire with high permeability for the presented method in this work.

### Symmetric response to voltage bias

**Figure 5:**
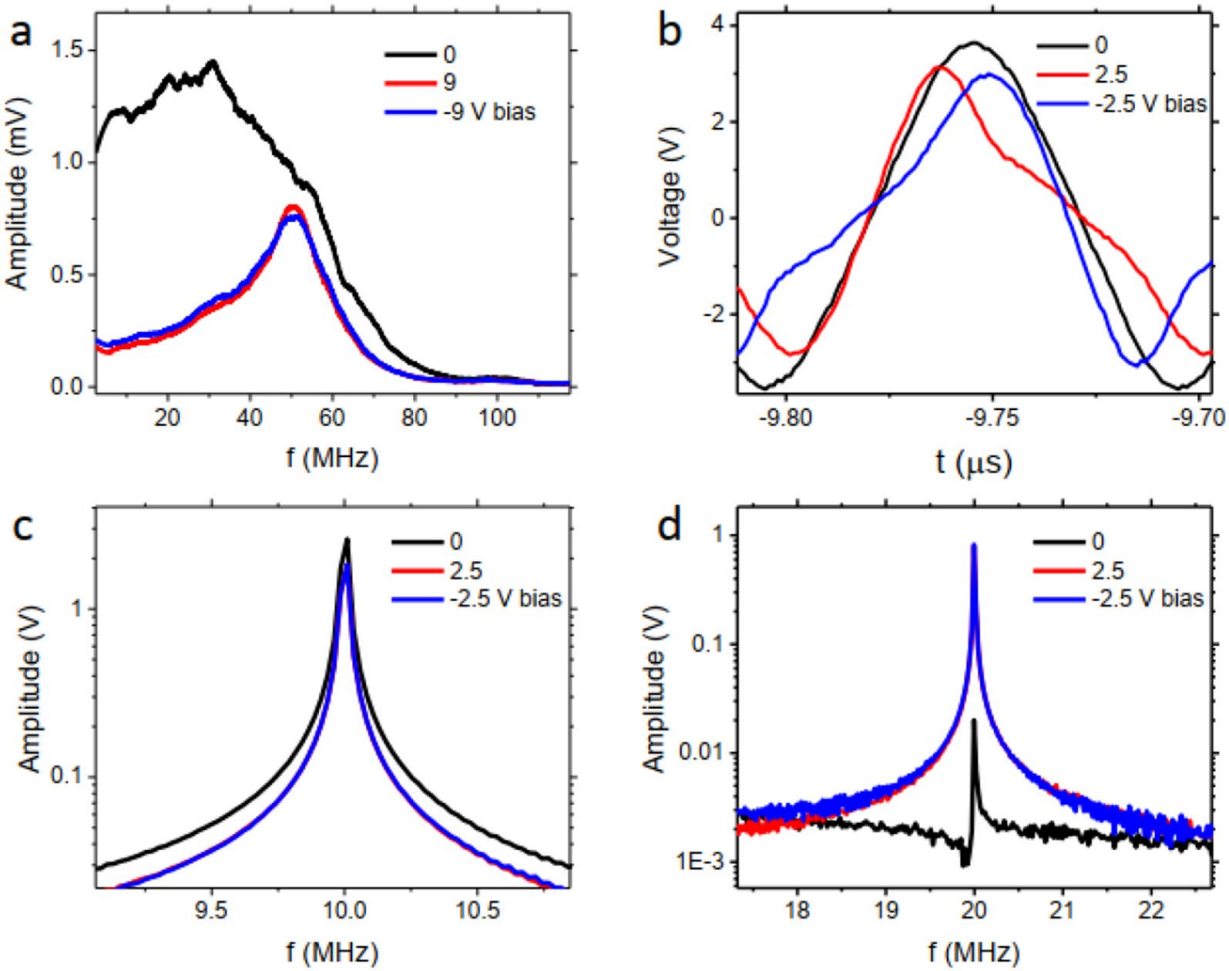
(**a**) FFT of the voltage signal of the magnetic wire under ± 9 V bias. (**b**) Voltage signal of the magnetic wire measured under ± 2.5 V bias voltage. FFT of the magnetic wires (**c**) first harmonic and (**d**) second harmonic voltage signal under ± 2.5 V bias voltage.

Another property of the wire and consequently of its electronic properties, is the symmetric structure and signal response. We applied a negative bias voltage to the system and observed a similar trend for the FFT peak frequency and amplitude shift. The FFT of the noise signal for ±9 V bias voltage are completely overlapped (Fig. 5a). The voltage signal of the oscilloscope for a ±2.5 V bias voltage (Fig. 5b) indicates asymmetric phase shifts for the higher harmonics. However, for voltage amplitudes, higher harmonics show the same behaviour for positive and negative bias voltage as the odd and the even harmonics amplitude (Fig. 5c,d, respectively) are overlapped for ±2.5 V bias.This observed symmetry is related to the alignment of the Oersted field, originating from the bias voltage, and the alignment of the domains in the wire. The wire with transverse magnetic anisotropy has parallel alignment of domains with the applied Oersted field, either positive or negative voltage. This similar geometry for domains alignment and the Oersted field in both positive and negative voltage biases results in a similar change in the magnetic permeability and therefore similar changes of the amplitude change. This result is observed for other voltage bias amplitudes as well and can be considered in circuit designs for a realistic computation.

## Possible circuit implementation for neuromorphic computing

**Figure 6:**
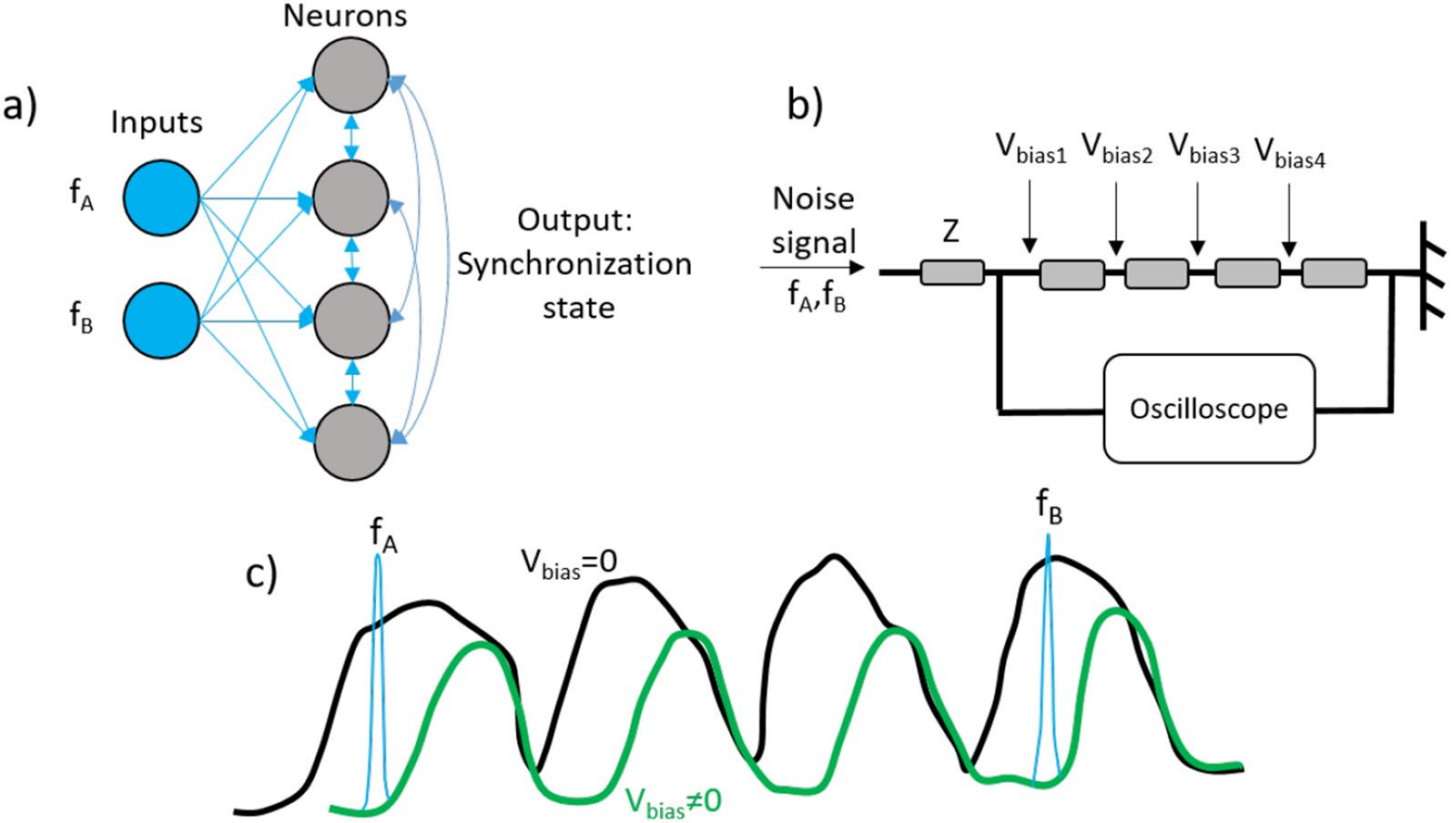
(**a**) Schematic of the neural network. (**b**) The implanted neural network equivalent to the neural network presented in panel (**a**). (**c**) The schematic of produced voltage amplitude in the frequency spectrum obtained from the oscilloscope in panel (**b**).

In this section, we explain a possible circuitry implementation for a neuromorphic computing-based task, i.e. vowel recognition. Figure 6a represents the schematic of a neural network where the output of each neuron can affect the other neurons (depicted via double arrow connections). Figure 6b demonstrates a circuitry schematic, equivalent to the neural network of Fig. 6a. Each wire can be set with specific material parameters so that the impedance peak of each wire in the frequency spectrum, schematized in Fig. 6c, are distinguishable. Noise signal and frequency characteristics of the vowel, encoded in $f_A$ and $f_B$, are applied to all the wires through an element with impedance Z. According to the wires characteristics, a shift of spectrum from black curve to the green curve is possible via application of $V_{DC}$ to the wires, providing the possibility of synchronizing the wires peak signal and the vowel characteristic frequencies which can be used for recognition of the vowel. Application of the $V_{DC}$ to the wires to achieve the synchronization between the vowel frequency characteristics and the wires peak spectrum is the way of training the network.

The work of Romera et al.^9^ is a successful demonstration of vowel recognition with spintronic devices. Much higher frequencies can be achieved in STOs, compared to the high permeability wires used in this work. In such spintronic-based devices, it has been anticipated to reach higher functioning frequencies to compensate the small number of involved elements in neuromorphic computing system. An example of these efforts is the theoretical work of Khymyn et al. ^31^ on antiferromagnetic oscillators where higher functioning frequencies (1 THz) are achievable. Despite the much lower functioning frequencies of the used wire in this work, we emphasis on the potential of the used system at high frequencies (GHz range) too^32^. A. Moya et al.^32^ have demonstrated the antenna behavior of Co-base wire with high permeability at GHz frequency range, indicating the potential of implementing the proposed approach based on environmental noise. This characteristic might open the pathway towards achieving a zero power computing system. Considering the possibility for device implementation, also we should mention the successful implementation of on-chip-integrated MI sensors which provides complementary metal-oxide semiconductor (CMOS) technology compatibility^33^. Therefore, our method can be applied to wires with much smaller dimensions which can provide lower voltage application possibility.

## Conclusion

We used frequency dependence of magnetoimpedance in a wire under noise signal to generate a voltage peak in the frequency spectrum. The presented tunability of the peak frequency and amplitude under bias voltage could be used in designs of neuromorphic computing systems. We suggest the implementation of some magnetic wires in a circuit to accomplish synchronisation through the application of a bias voltage to each wire. The possibility of coupling our magnetic wires through magnetic dipole/exchange interaction and also electrical voltage/current gives the opportunity of training for a neural network-based computing system. The known hysteretic and SOT-driven MI response of the wires can add up to the promises of the presented method. We suggest the application of this formalism to systems which show frequency dependent response.

## Supplementary Information

Supplementary Information.

## Data Availability

The datasets generated during and/or analysed during the current study are available from the corresponding author on reasonable request.

## References

[1] [Editorial]. Computing on the brain. *Nat. Electron.* **3**, 347 (2020).

[2] Misha Rabinovich GL, Huerta R (2008). Neuroscience: Transient dynamics for neural processing. Science.

[3] Sussillo D (2014). Neural circuits as computational dynamical systems. Curr. Opin. Neurobiol..

[4] Pikovsky A, Rosenblum M (2015). Dynamics of globally coupled oscillators: Progress and perspectives. Chaos.

[5] Borisyuk R, Denham M, Hoppensteadt F, Kazanovich Y, Vinogradova O (2000). An oscillatory neural network model of sparse distributed memory and novelty detection. BioSystems.

[6] Raychowdhury A (2019). Computing with networks of oscillatory dynamical systems. Proc. IEEE.

[7] Bressan F, Hess RL, Sgarbossa P, Bertani R (2019). Chemistry for audio heritage preservation: A review of analytical techniques for audio magnetic tapes. Heritage.

[8] Cros, V., Fert, A., Sénéor, P. & Petroff, F. The 2007 nobel prize in physics: Albert Fert and Peter Grünberg. In *The Spin. Progress in Mathematical Physics* Vol. 55 (eds Duplantier, B. *et al.*) (Birkhäuser, 2009).

[9] Romera M (2018). Vowel recognition with four coupled spin-torque nano-oscillators. Nature.

[10] Torrejon J (2017). Neuromorphic computing with nanoscale spintronic oscillators. Nature.

[11] Zahedinejad M (2020). Two-dimensional mutually synchronized spin Hall nano-oscillator arrays for neuromorphic computing. Nat. Nanotechnol..

[12] Chiolerio A (2007). Thermally evaporated Cu–Co top spin valve with random exchange bias. J. Appl. Phys..

[13] Gregg J (2003). Spin injection efficiency in spin electronic devices. J. Magn. Magn. Mater..

[14] Chiolerio, A. Spintronic Devices. Ph.D. thesis, Politecnico di Torino (2009).

[15] Yelon A, Britel M, Ménard D, Ciureanu P (1997). Origin of linear and nonlinear giant magnetoimpedance. Physica A Stat. Mech. Appl..

[16] Dufay B (2017). Low frequency excess noise source investigation of off-diagonal GMI-based magnetometers. IEEE Trans. Magn..

[17] Knobel M, Pirota KR (2002). Giant magnetoimpedance: Concepts and recent progress. J. Magn. Magn. Mater..

[18] Zhukova V (2002). Optimization of giant magnetoimpedance in Co-rich amorphous microwires. IEEE Trans. Magn..

[19] Harrison EP, Turney GL, Rowe H (1935). Electrical properties of wires of high permeability. Nature.

[20] Hajiali MR (2017). Spin-orbit-torque driven magnetoimpedance in Pt-layer/magnetic-ribbon heterostructures. Appl. Phys. Lett..

[21] Dolabdjian C, Dufay B, Saez S, Yelon A, Ménard D (2014). Is low frequency excess noise of GMI induced by magnetization fluctuations?. Mater. Appl. Sens. Transducers III.

[22] Jamilpanah L (2017). Magnetoimpedance exchange coupling in different magnetic strength thin layers electrodeposited on Co-based magnetic ribbons. J. Phys. D Appl. Phys..

[23] Hu L (2023). Soft magnetic properties and giant magneto-impedance effect of Co$$_68.15$$Fe$$_4.35$$Si$$_12.5$$B$$_15-x$$Cr$$_x$$ amorphous ribbons. J. Non-Cryst. Solids.

[24] Zhang SL, Sun JF, Xing DW, Fang DQ, Wang LC (2013). Frequency dependence of magnetization and giant magneto impedance effect of amorphous wires. Int. J. Miner. Metall. Mater..

[25] Seddaoui D, Ménard D, Ciureanu P, Yelon A (2007). Second harmonic of nonlinear magnetoimpedance in amorphous magnetic wires with helical anisotropy. J. Appl. Phys..

[26] Gémez-Polo C, Vázquez M, Knobel M (2001). Rotational giant magnetoimpedance in soft magnetic wires: Modelization through Fourier harmonic contribution. Appl. Phys. Lett..

[27] Clime L (2004). Non-linear magetoinductance in amorphous wires. Physica B Condens. Matter.

[28] Gómez-Polo C, Duque JGS, Knobel M (2004). Nonlinear giant magnetoimpedance and the asymmetric circumferential magnetization process in soft magnetic wires. J. Phys. Condens. Matter.

[29] Duque JGS (2004). The effect of helical magnetoelastic anisotropy on magnetoimpedance and its second harmonic component in amorphous wires. J. Magn. Magn. Mater..

[30] Buznikov NA, Antonov A, Rakhmanov A (2010). Effect of direct current on higher harmonic generation in the frequency spectrum of magnetoimpedance of amorphous wires with circular anisotropy. Tech. Phys..

[31] Khymyn R, Lisenkov I, Voorheis J, Sulymenko O, Prokopenko O, Tiberkevich V, Akerman J, Slavin A (2018). Ultra-fast artificial neuron: Generation of picosecond-duration spikes in a current-driven antiferromagnetic auto-oscillator. Sci. Rep..

[32] Moya A, Archilla D, Navarro E, Hernando A, Marín P (2019). Scattering of microwaves by a passive array antenna based on amorphous ferromagnetic microwires for wireless sensors with biomedical applications. Sensors.

[33] Karnaushenko D (2015). Self-assembled on-chip-integrated giant magneto-impedance sensorics. Adv. Mater..

