# Supplementary material for: Proposing magnetoimpedance effect for neuromorphic computing
Source: Sci Rep. 2023 May 27;13:8635. doi: 10.1038/s41598-023-35876-0 (PMC10224953; doi:10.1038/s41598-023-35876-0)
Supplement: Supplementary file 1 — Supplementary Information. [file 41598_2023_35876_MOESM1_ESM.docx]

Supplementary information

Proposing magnetoimpedance effect for
neuromorphic computing

Loghman Jamilpanah^1,2,^*, Alessandro Chiolerio^3,4^, Marco Crepaldi^4^, Andrew Adamatzky^5^, Majid Mohseni^1,^**

^1^Faculty of Physics, Shahid Beheshti University, Evin, 19839 Tehran, Iran

^2^Laboratory for High Performance Ceramics, Empa, Swiss Federal Laboratories for Materials Science and Technology, Uberlandstrasse 129, Dübendorf, CH - 8600, Switzerland

^3^Bioinspired Soft Robotics, Center for Converging Technologies, Istituto Italiano di Tecnologia, Via Morego 30, 16165 Genova, Italy

^4^Electronic Design Laboratory, Center for Human Technologies, Istituto Italiano di Tecnologia, Via Enrico Melen 83, 16152 Genova, Italy

^5^Unconventional Computing Laboratory, University of the West of England, Coldharbour Lane, BS16 1QY Bristol, UK

^*,**^Correspondence: [loghmanjamilpanah@gmail.com](mailto:loghmanjamilpanah@gmail.com), m_mohseni@sbu.ac.ir

**Simulation of the harmonics signal**

Simulations of the magnetic wire *per se* and of wires connected in series, parallel and ladder, were performed using Matlab R2021b in the Simulink environment, under Simscape Electrical. Each wire is modelled with the parallel of a resistor (the real component of the wire impedance, modelled including thermal noise at 25 °C) and an inductor (the imaginary component of the wire impedance, featuring Gaussian random tolerance and initial inductor current of 100 mA), the noise is modelled using a voltage source featuring the superposition of a DC bias (0.1;1 and 10V) and an AC noise (0.1V; 10 MHz).

Numerical simulations performed under the Simscape environment of a single wire (see Figure S1) offer a picture perfectly in line with experimental observations, with an additional information about higher DC biases that can suppress higher harmonic oscillations. This mechanism could be used as a trigger. The configuration of multiple magnetic wires in parallel is shown to be less sensitive to higher harmonics suppression, in comparison to the series connection, where the higher harmonics appear only for the smallest biases (0.1 V). Finally, the ladder configuration provides the best tuneability of higher harmonic suppression: the smallest bias of 0.1 V allows to observe 22 resonances in the range up to 50 MHz; the intermediate bias of 1 V allows to observe 9 of them; the highest bias of 10 V suppresses all of the superior harmonics, leaving only the noise signal at 10 MHz.


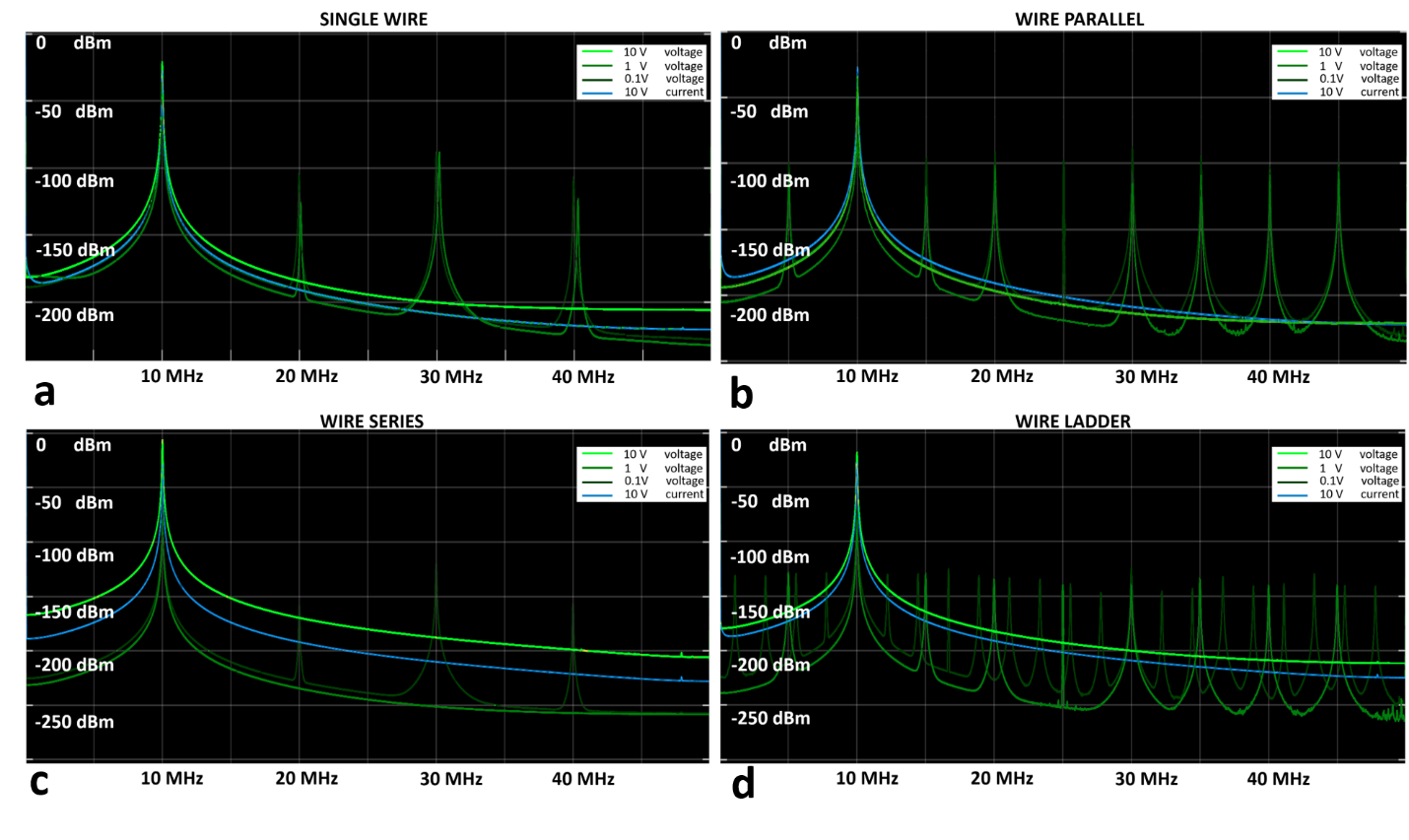


**Figure S1.** a) Single wire under different DC biases and 10 MHz noise signal. b) Parallel of five wires under different DC biases and 10 MHz noise signal. c) Series of five wires under different DC biases and 10 MHz noise signal. d) Ladder of five wires under different DC biases and 10 MHz noise signal.
